# The interactions between ineffective erythropoiesis and ferroptosis in β-thalassemia

**DOI:** 10.3389/fphys.2024.1346173

**Published:** 2024-02-26

**Authors:** Siyang Lin, Yanping Zheng, Meihuan Chen, Liangpu Xu, Hailong Huang

**Affiliations:** ^1^ Fujian Provincial Key Laboratory of Prenatal Diagnosis and Birth Defect, Medical Genetic Diagnosis and Therapy Center of Fujian Maternity and Child Health Hospital College of Clinical Medicine for Obstetrics and Gynecology and Pediatrics, Fujian Medical University, Fuzhou, China; ^2^ The School of Medical Technology and Engineering, Fujian Medical University, Fuzhou, China; ^3^ Fujian Clinical Research Center for Maternal-Fetal Medicine, Fuzhou, China; ^4^ National Key Obstetric Clinical Specialty Construction Institution of China, Fuzhou, China

**Keywords:** β-thalassemia, ineffective erythropoiesis, ROS, ferroptosis, iron overload, pathogenesis

## Abstract

In Guangxi, Hainan, and Fujian Province in southern China, β-thalassemia is a frequent monogenic hereditary disorder that is primarily defined by hemolytic anemia brought on by inefficient erythropoiesis. It has been found that ineffective erythropoiesis in β-thalassemia is closely associated with a high accumulation of Reactive oxygen species, a product of oxidative stress, in erythroid cells. During recent years, ferroptosis is an iron-dependent lipid peroxidation that involves abnormalities in lipid and iron metabolism as well as reactive oxygen species homeostasis. It is a recently identified kind of programmed cell death. β-thalassemia patients experience increased iron release from reticuloendothelial cells and intestinal absorption of iron, ultimately resulting in iron overload. Additionally, the secretion of Hepcidin is inhibited in these patients. What counts is both ineffective erythropoiesis and ferroptosis in β-thalassemia are intricately linked to the iron metabolism and Reactive oxygen species homeostasis. Consequently, to shed further light on the pathophysiology of β-thalassemia and propose fresh ideas for its therapy, this paper reviews ferroptosis, ineffective erythropoiesis, and the way they interact.

## 1 Introduction

Thalassemia, a frequent monogenic hereditary disorder, consists mainly of α-thalassemia and β-thalassemia (β-thal). Point mutations or deletions of the β-globin gene cluster, which is located on the human chromosome 11 p15.3 locus, induce β-thal pathogenicity by reducing or eliminating β-globin synthesis (Taher et al., 2018). Hemolytic anemia is the main pathological manifestation of β-thal, and hemolysis occurs for two reasons: premature destruction of erythroid precursor cells, and shortening of the lifespan of mature erythrocytes in the circulation (Fibach and Dana, 2019). At present, β-thal is mainly treated with blood transfusion, iron chelation, stimulation of fetal hemoglobin synthesis, bone marrow transplantation, and gene therapy (Ali et al., 2021).

Anemia in individuals who have β-thal is primarily triggered by ineffective erythropoiesis (IE). Research on the erythrocyte and iron kinetics in β-thal sufferers indicates that around 65% of nucleated erythrocytes perish before maturation (Pootrakul et al., 2000). Reactive oxygen species (ROS) deposition is a significant driver of, IE as well as one of its effects, which can cause oxidative stress in the erythroid cells. Excessive ROS in β-thal is mainly produced by mitochondrial impairment due to chronic anemia caused by, IE (Tyan et al., 2014).

The concept of ferroptosis, a recently identified kind of programmed cell death, was introduced in 2012 by Brent R. Stockwell’s team. Ferroptosis is an iron-dependent lipid peroxidation that involves imbalances in lipid homeostasis, iron homeostasis, and ROS homeostasis (Fuhrmann and Brüne, 2022). Since ferroptosis is caused by an excess of iron, iron’s involvement in the disease is undeniable (Mancardi et al., 2021). Furthermore, iron overload is one of the important complications of β-thal, which can be caused by exogenous blood transfusion and, IE. Given the increased research on, IE and ferroptosis in β-thal, this review aims to comprehensively summarize the intricate interplay between, IE and ferroptosis in β-thal, to shed further light on the pathophysiology and propose fresh ideas for its therapy.

## 2 IE in β-thal

**FIGURE 1:**
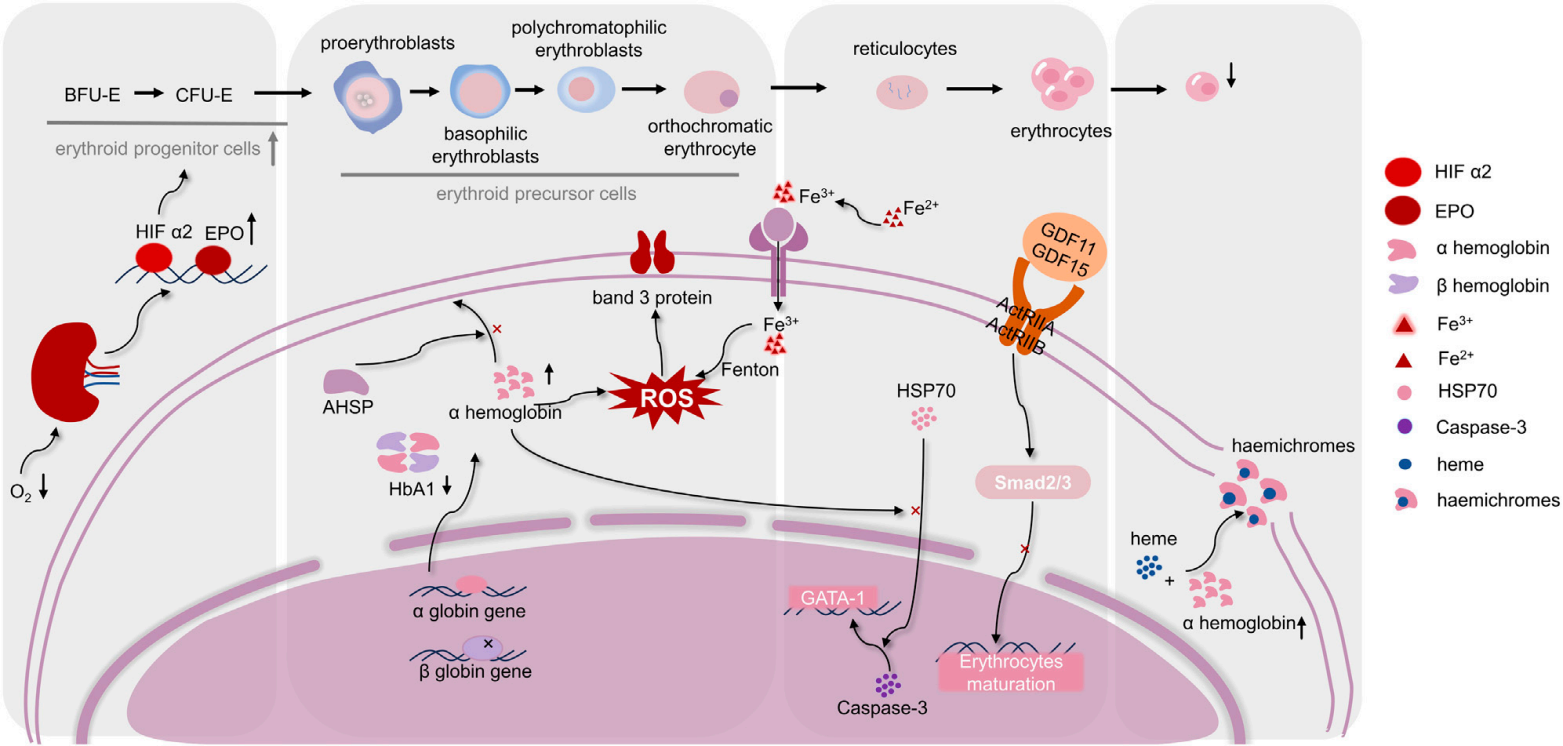
Regulation mechanisms of differentiation and maturation of erythroid cells in, IE. The maturation of erythrocytes needs to go through hematopoietic stem cells, erythroid progenitor cells, erythroid precursor cells, and erythrocytes stages. As shown in the figure, when, IE occurs, various stages of differentiation and maturation of erythroid cells are respectively affected by several molecular mechanisms. Moreover, ROS homeostasis is in a key position in the occurrence of, IE, which connects the molecular mechanisms at each stage. (Abbreviations: BFU-E, burst-forming unit-erythroid; CFU-E, colony forming unit-erythrocyte; HIF α2, hypoxia-inducible factorα2; EPO, Erythropoietin; AHSP, α-hemoglobin stabilizing protein; HbA1, hemoglobin A1; HSP70, heat shock protein 70; ROS, reactive oxygen species; GDF11, 15, growth differentiation factor 11,15; ActRIIA, ActRIIB, activation of activin receptors IIA, IIB).

Aberrant erythroid cell maturation and differentiation are hallmarks of, IE, which is not the main cause of β-thal, but it keeps patients with β-thal in a harmful state. IE roughly undergoes four stages, starting with the expansion of erythroid progenitors, followed by accelerated erythroid differentiation to the polychromatic erythrocyte stage, followed by blockage of polychromatic erythrocyte maturation and, finally, an increase in polychromatic erythrocyte death (Arlet et al., 2016). Interestingly, a number of scholars have studied the mechanism of four stages of ineffective erythropoiesis, and studies have shown that iron metabolism and ROS homeostasis are crucial in all four of them (Figure 1).

### 2.1 The main causes of IE

#### 2.1.1 Abnormal proliferation and differentiation of erythroid hematopoietic progenitor cells

The differentiation and maturation of erythroid cells involves several stages. Firstly, pluripotent hematopoietic stem cells proliferate and differentiate into erythroid hematopoietic progenitor cells. Subsequently, the erythroid hematopoietic progenitor cells differentiate into the stages of proerythrocytes, early erythroblasts, intermediate erythroblasts, late erythroblasts, and reticulocytes. Finally, reticulocytes enucleate to form mature erythrocytes (Caulier and Sankaran, 2022). Increasing studies showed that **e**rythroid hematopoietic progenitor cells exhibit a noteworthy increase in cell proliferation in β-thal patients (Ribeil et al., 2013). According to Ramos, an intermediate β-thal mouse model reveals the development of erythroid hematopoietic progenitor cells, with the expansion being significantly more prominent in the spleen than in the bone marrow (Ramos et al., 2013). This was attributed to hypoxia in the tissues of patients with β-thal, which stimulates hypoxia-inducible factor α2 to enhance erythropoietin (EPO) production (Huang et al., 2019). EPO serves as a crucial driver for erythropoiesis, and its excessive production is anticipated to augment the amount of mature erythrocytes by raising the percentage of erythroid hematopoietic progenitor cells (Gupta et al., 2018). This compensatory mechanism leads to extramedullary hematopoiesis, and damaged erythroblasts are cleared by macrophages and reticuloendothelial system within the splenic sinusoids, ultimately causing some patients with β-thal to present with splenomegaly.

#### 2.1.2 Abnormal differentiation of erythroid precursor cells

Erythroid precursor cells encompass cells at various stages ranging from proerythroblasts to orthochromatic erythrocytes. Although the production of erythroid progenitor cells is increased in β-thal in response to EPO, the demise of erythroid precursor cells remains inevitable. The reduction or absence of β-globin within the erythroblasts of those with β-thal causes a discrepancy in the proportion of α-globin and β-globin over the development of erythroid cells. Additionally, excess α-protein is deposited on the cell membranes, damaging the erythroid precursor cells and ultimately resulting in anemia (Higgs et al., 2012; Cazzola, 2022). There is a scavenger protein in normal erythroblasts, called α-hemoglobin stabilizing protein (AHSP), which can effectively prevent cell membrane damage caused by small amounts of α-globin protein precipitation (Han et al., 2022). Notably, the latest study has found that AHSP is not sufficient to control excess excessive levels of α-globin in β-thal (Che Yaacob et al., 2020). As a result, erythroid precursor cells in β-thal have increased death and are cleared by macrophages and the reticuloendothelial system.

#### 2.1.3 Abnormal maturation of erythroblasts

During the maturation of erythroid cells, alterations in proteins and cytokines are intricately linked to, IE. Glutathione peroxidase 4 (GPX4), an antioxidant enzyme, has been reported to be required for the maturation of erythroid cell. Decreased expression of GPX4 leading to hemolytic anemia and increased splenic erythroid progenitor cells death was observed in mouse hematopoietic cells, and Altamura also noted that low expression of GPX4 corresponds to reticulocyte maturation problem (Altamura et al., 2020). It is unclear how GPX4 influences erythropoiesis, although the research indicated that it can influence nucleus extrusion by performing a part in the lipid raft organization (Ouled-Haddou et al., 2020).

The transient activation of cysteine aspartate specific protease-3 (Caspase-3) is necessary for erythroblasts maturation, and GATA-1 is also essential for the maturation of erythroblasts (Dong et al., 2020). In the terminal stage of normal erythroid cells differentiation and maturation, Heat shock protein 70 (HSP70) is translocated from the cytoplasm into the nucleus, and it can serve as a chaperone protein within the nucleus to shield the GATA-1 from being cleaved by Caspase-3 (Ribeil et al., 2007; Arlet et al., 2014). The localization of HSP70 is regulated by the export protein-1, which regulates the export of HSP70 from the nucleus to the cytoplasm. However, α-globin amassment in β-thal leads to HSP70 sequestration within the cytoplasm, compromising its protective effect on GATA-1 and ultimately impeding erythroblasts maturation (Guillem et al., 2020).

Another contributing factor for erythroblasts maturation is the presence of growth differentiation factor 11 (GDF11) and 15 (GDF15), which belong to the transforming growth factor-β (TGF-β) superfamily and influence erythroid maturity by modulating the Smad2/3 signaling pathway. In addition, it has been suggested that GDF11 and GDF15 inhibit erythroid maturation through activation of activin receptors IIA (ActRIIA) and IIB (ActRIIB) (Suragani et al., 2014b). According to research by Ranjbaran, GDF15 expression progressively rises through late-stage erythroid division and negatively regulates erythroblasts growth, development, and proliferation of proliferation of erythrocytes (Ranjbaran et al., 2020). Furthermore, Dussiot revealed that the RAP-536 and RAP-011, ligands for activin receptor IIa, exhibit potential in ameliorating, IE in a mouse model of β-thal (Dussiot et al., 2014). What is more, the GDF11-mediated Smad2/3 signaling pathway is substantially repressed by RAP-536, which binds to GDF11 and promotes the maturation of late erythroid precursor cells (Suragani et al., 2014a). RAP-011 may alleviate anemia symptoms associated with β-thal by mitigating the detrimental effects caused by GDF11 through mechanisms involving the mitigation of cellular oxidative damage and the prevention of α-globin precipitation.

#### 2.1.4 Reduced lifespan of mature erythrocytes

HbA1, a tetramer made up of two α- and two β-globin chains, is the most prevalent kind of hemoglobin in healthy adults. According to the production of the β-protein chain, patients with β-thal can be classified into β^+^-thalassemia (reduced β-globin chain synthesis) and β^0^-thalassemia (complete inability to synthesize β-globin chain) (Jaing et al., 2021). The reduction or absence of β-globin leads to an imbalance between α-globin and β-globin, and excess α-globin accumulates in erythrocytes, resulting in a tendency for excess unstable α-globin chains to bind heme, forming highly insoluble α-globin inclusions (haemichromes), which are deposited on cell membranes, altering the permeability of the erythrocyte membranes (Higgs et al., 2012; Longo et al., 2021). Alterations in erythrocyte membrane permeability lead to a decrease in the efficiency of ATP production and a reduction in the total lifespan of erythrocytes, inducing erythrocyte death, which contributes to, IE and heightened hemolysis (Zahedpanah et al., 2014).

### 2.2 The effect of severe ROS buildup in IE

A by-product of living organisms’ ordinary oxygen metabolism is ROS. It is extremely important in maintaining homeostasis and signaling within cells. On the other hand, ROS may seriously fry cells when their levels climb abruptly, which is known as oxidative stress (Yang and Lian, 2020). Erythroid cells primarily generate ROS through enzymatic reactions and non-enzymatic reactions. Activation of NADPH oxidase and hemoglobin auto-oxidation contribute significantly to the substantial intracellular ROS production in erythroblasts (Bettiol et al., 2022). The dynamic fluctuations in ROS amounts in erythroid cells throughout various growing phases have been demonstrated in previous research. The erythroblasts of β-thal exhibit a greater amount of ROS contrasted with standard erythroid precursor cells, especially in the subsequent steps of erythroid maturity. Therefore, the overproduction of ROS adversely impacts the differentiation and maturation processes of erythroid cells (Fibach and Dana, 2019; Yang et al., 2023). The deleterious impact of ROS accumulation on hematopoietic progenitor cells was conducted in another work by Ludin, thereby highlighting the significant role of ROS accumulation on, IE (Ludin et al., 2014).

The main causes of excess production of ROS in β-thal are the accumulation of α-globin and iron overload. On one hand, excess unstable α-globin chains bind with heme to form haemichromes that are deposited in the erythroblasts’ membrane. These haemichromes are extremely hazardous and may initiate the production of ROS, including hydroxyl radicals, thereby causing oxidative damage in erythroid cells (Matte and De Franceschi, 2019). The ROS generated by haemichromes on the erythroblasts’ membrane poses a challenge to removal by the cytoplasmic antioxidant system, and readily oxidizes lipid and protein constituents of the cell membrane, leading to severe oxidative damage. On the other hand, β-thal is characterized by iron excess, provoking the Fenton reaction to produce ROS, certainly impacting, IE (Gupta et al., 2018). Moreover, iron serves as a vital cofactor for oxidoreductases in the mitochondrial electron transport chain and generates excess ROS through this electron transport chain (Read et al., 2021).

As the most representative protein in the erythroblasts’ membrane, twenty-five percent of the membrane proteins are composed of the human band 3 protein. It is known as the “anion channel” and has two structural domains, a transmembrane domain and a cytoplasmic domain, which are involved in transmembrane information transfer and the management of the development and differentiation of cells (Remigante et al., 2021). One possible mechanism is that ROS accumulation due to aberrant oxidative stress in erythroid cells affects erythropoiesis by inducing oxidative denaturation of the band 3 protein. Oxidative denaturation of the protein reduces the erythroblasts membrane’s deformability and makes it more susceptible to clearance by immune organs such as the spleen, ultimately leading to anemia (Pantaleo et al., 2008).

According to current studies, several molecular mechanisms may be involved in the promotion of ROS overproduction in erythrocytes by the oxidative system. Firstly, in the Hbbth3/+ mouse model of β-thal, 20-hydroxyeicosatetraenoic acid, a metabolite of Cytochrome P450 4A/F (CYP4A/F), mediates ROS overproduction through a NADPH-dependent pathway (Bou-Fakhredin et al., 2021). Secondly, the generation of ROS and, IE is stimulated by the downregulation of isocitrate dehydrogenase 1 along with an increase in α-ketoglutarate (Gonzalez-Menendez et al., 2021). Finally, miRNA has been confirmed to regulate ROS levels and consequently impact, IE. For instance, aberrant expression of miR-9 can inhibit the expression of Forkhead box O3, an erythroid transcription regulator, leading to increased ROS levels and impaired erythropoiesis (Zhang et al., 2018). Besides, miR-214 affects the oxidative damage of erythroid cells by regulating the level of activating transcription factor 4, and a positive correlation exists between the expression degree and ROS levels., thereby affecting the production of erythrocytes (Saensuwanna et al., 2021).

## 3 Ferroptosis

**FIGURE 2:**
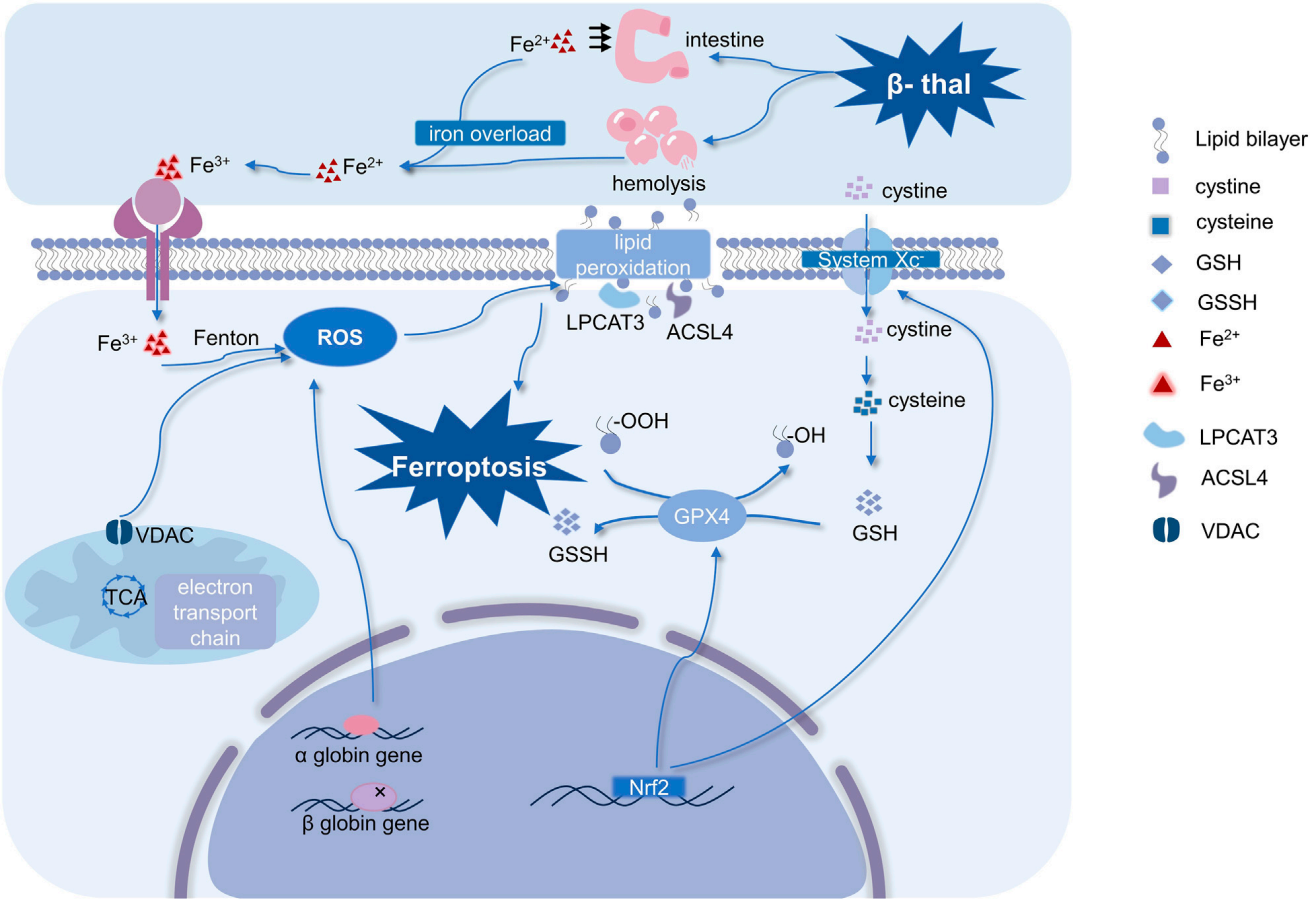
Mechanisms of ferroptosis in β-thal and occurrence and regulation of ferroptosis. Ferroptosis in β-thal is thought to be caused by excessive iron-dependent ROS production, and it is driven by iron-dependent lipid peroxidation. Therefore, ferroptosis is characterized by an imbalance in iron homeostasis and ROS homeostasis. It is interesting to note that mitochondria are crucial for controlling ROS homeostasis. Moreover, system Xc^−^, Nrf2, and GPX4 are the primary regulators of ferroptosis. (Abbreviations: VDAC, voltage-dependent anion channel; ROS, reactive oxygen species; GSSH, oxidized glutathione; GSH, glutathione; Nrf2, nuclear factor erythroid 2-related factor 2; GPX4, glutathione peroxidase 4; LPCAT3, lysophosphatidylcholine acyltransferase-3; ACSL4, Acyl-CoA synthetase long-chain family member 4; β-thal, β-thalassemia; TCA, tricarboxylic acid cycle).

Ferroptosis can be suppressed by lipid peroxidation inhibitors, which is thought to be triggered by lipid peroxidation that depends on iron (Capelletti et al., 2020; Jiang et al., 2021). The morphological manifestation of ferroptosis is distinguished by diminished mitochondrial cristae, atrophy of entire mitochondria, normal size of the nucleus, and intact cell membrane. Functionally, ferroptosis serves as an essential physiological mechanism for the maintenance of homeostasis in the body’s internal environment, as well as a pathological mechanism underlying the onset and development of human diseases. Growing evidence suggests that ferroptosis is present in various disorders, and it plays a very different role in different diseases (Liu et al., 2022). For examples, promoting ferroptosis in cancer cells is beneficial for cancer treatment, however, ferroptosis is one of the pathophysiological mechanisms in neurodegenerative disorders (Lei et al., 2022; Ryan et al., 2023). Initially, the value of ferroptosis in aging and embryonic erythropoiesis was established, and Somanathapura determined that heme-mediated ferroptosis could be crucial in hemolytic disorders (NaveenKumar et al., 2018; Zheng et al., 2021). β-thal, a kind of hemolytic anemia, is closely related to abnormal embryonic erythropoiesis, thus, its pathogenesis may be probably highly linked to ferroptosis. The mechanisms of ferroptosis related to β-thal and the occurrence and regulation of ferroptosis are summarized in Figure 2.

### 3.1 Mechanisms of ferroptosis

#### 3.1.1 Iron overload is an important cause of ferroptosis

Iron overload is a pivotal component in the occurrence of ferroptosis. The addition of exogenous iron heightened HT-1080 cells’ susceptibility to ferroptosis inducers (Dixon et al., 2012). Notably, several iron-containing enzymes such as 12-lipoxygenase and Cytochrome P450 oxidoreductase are known to have a promotional role in facilitating lipid peroxidation, thereby driving ferroptosis (Stockwell, 2022). While maintaining appropriate levels of iron is vital for regular physiological activities, excessive accumulation can cause cellular ferroptosis (Li et al., 2020). The above evidence underscores the critical involvement of iron in mediating ferroptosis.

Iron metabolism is mainly regulated by substances secreted by the liver such as Transferrin (TF), Transferrin receptor (TFR), Ferritin and Hepcidin. They are vital in maintaining systemic iron equilibrium (Rochette et al., 2022). With a molecular weight of roughly 80 kDa, TF is a glycoprotein generated in the liver and transported into the bloodstream. It contains two specific high-affinity binding sites that bind Fe^3+^ and transport them to various tissues and organs (Kawabata, 2019). TFR acts as a receptor ligand for TF. In normal iron metabolism, Fe^2+^ released from intestinal absorption as well as erythrocyte destruction is oxidized to Fe^3+^ by metal oxidases, and the TF-Fe^3+^ complex formed by the binding of Fe^3+^ to TF, which binds to TFR1 and enters the cells (Berdoukas et al., 2015). Gao’s study revealed that TF serves as an inducer of ferroptosis, and TFR exhibits a close association with this process (Gao et al., 2015). Furthermore, ferritin also exerts a regulatory effect on ferroptosis. A prominent kind of iron-storing protein, ferritin is composed of 24 subunits, the light chain (FTL) and heavy chain (FTH) of which may store up to 4,500 iron atoms apiece (Plays et al., 2021). By increasing free iron through ferritin autophagy, a selective autophagy mechanism that targets ferritin, ferritin might encourage ferroptosis (Li et al., 2022). Hou demonstrated that autophagy induces ferroptosis through the degradation of ferritin in fibroblasts and cancer cells (Hou et al., 2016). However, when excess iron continues to accumulate in the body beyond the transport capacity of TF saturation (>70%), Non-Transferrin Bound Iron (NTBI) and Labile Plasma Iron (LPI) levels are elevated, leading to the formation of unstable intracellular labile iron pools (LIP) and causing iron overload.

#### 3.1.2 ROS imbalance plays an important role in ferroptosis

Lipid peroxidation is the fundamental root cause of ferroptosis, and since lipids make up the majority of cell membranes, they are also the dominating targets of ROS. It causes an imbalance in the cellular oxidative homeostasis, which in turn triggers oxidative damage and ferroptosis. The Malondialdehyde and 4-hydroxynonenal acid are the end products of lipid peroxidation, which can be driven by enzymatic reactions. The specific mechanism by which enzymatic reactions cause lipid peroxidation is that Acyl-CoA synthetase long-chain family member 4 (ACSL4) and Lysophosphatidylcholine acyltransferase-3 (LPCAT3) first activate polyunsaturated fatty acids (PUFAs) to bind to lipids in the cell membrane (e.g., phosphatidylethanolamine PE), forming the PUFA-PE complex (Su et al., 2019). Subsequently, the PUFA-PE complex undergoes catalysis by lipoxygenases (LOXs) and cyclooxygenases (COXs), resulting in lipid peroxidation (Shah et al., 2018). Non-heme iron-containing dioxygenases, or LOXs, have been demonstrated to specifically target and oxidize PUFAs, hence promoting lipid peroxidation ultimately triggering ferroptosis (Wang et al., 2021).

Morphological alterations to the mitochondria occur during ferroptosis when atrophy of the whole mitochondria and a decrease in mitochondrial cristae are seen. Concurrently, the mitochondrial membrane potential also changes, which is mediated by the presence of a voltage-dependent anion channel (VDAC) on the membranes. Erastin works on mitochondrial VDAC and releases a significant quantity of ROS, which ultimately causes ferroptosis (Yagoda et al., 2007). Many metabolic pathways associated with mitochondria, such as the mitochondrial tricarboxylic acid cycle and electron transport chain, have the capability to generate ROS. Excessive ROS production leads to detrimental effects on mitochondrial proteins and lipids, causing oxidative damage and facilitating ferroptosis (Battaglia et al., 2020). Furthermore, it is also critical to keep in mind that aberrant iron metabolism, lipid peroxidation, and aberrant mitochondrial function all work together to release ROS, rather than each occurring alone.

The underlying cause of ferroptosis is a disequilibrium between the body’s oxidants and antioxidants. As an antioxidant enzyme, GPX4 may efficiently reduce lipid peroxidation (Ursini and Maiorino, 2020). Ferroptosis can be inhibited by boosting the expression of GPX4. Conversely, inhibition of GPX4 expression promotes ferroptosis. GPX4 converts glutathione (GSH) to oxidized glutathione (GSSG) and converts lipid peroxides to the corresponding alcohols, thus preventing the occurrence of Fenton reactions and subsequently inhibiting ROS generation (Forcina and Dixon, 2019). Hence, GPX4 acts a pivotal part in preserving the body’s oxidative equilibrium, restraining lipid peroxidation, and suppressing ferroptosis (Liu Y. et al., 2023).

A key component of the cellular antioxidant system, System Xc^−^ is a type of amino acid anti-transporter that is substantially dispersed in a lipid bilayer and is a very selective cystine uptake system (Liu et al., 2021). System Xc^−^ consists of two subunits, one is solute carrier family 7 member 11 (SLC7A11) and the other is solute carrier family 3 member 2 (SLC3A2), which enables the import of cystine from the extracellular space. Intracellular cystine is reduced to cysteine by cystine reductase, and the cysteine is utilized by Geosynthetic clay liner (GCL) and Glutathione synthetase (GSS) enzymes for GSH production (Seibt et al., 2019). Thus, any impairment to either of the system Xc^−^-glutathione-GPX4 axis compromises the conversion of lipid peroxides to the corresponding alcohols, ultimately hampering the GPX4 antioxidant effect and giving rise to ferroptosis (Chen et al., 2021).

Nuclear factor erythroid 2-related factor 2 (Nrf2) has been found to be a key regulator in inhibiting ferroptosis. Nrf2 is considered to be a key part of organismal antioxidants since several of Nrf2’s downstream genes—including the previously described system Xc^−^ and GPX4 are involved in reversing ferroptosis (Somparn et al., 2019). The downstream targets of Nrf2 can be classified into three categories, which are the regulation of iron metabolism, intermediary metabolism, and glutathione synthesis/metabolism (Dodson et al., 2019). Notably, Nrf2 governs various aspects of iron metabolism by regulating key players including FTL and FTH, which stores iron, and FPN, which transports iron. In addition, SLC7A11 and GCL have also been shown to be regulated by Nrf2(Kang et al., 2021). According to what is stated above, Nrf2 inhibits ferroptosis through multiple pathways.

### 3.2 Ferroptosis in HSCs

Hematopoietic stem cells (HSCs) are the source of human blood cells because they have the capacity to self-renew, self-repair, and rebuild hematopoietic function. The capacity to rebuild hematopoietic function refers to the ability to preserve and permanently restore normal hematopoietic function in the future. Furthermore, because HSCs are able to self-renew and self-repair, their replication is asymmetrical, with one daughter cell retaining all of the features of hematopoietic stem cells and the other daughter cell continuing to proliferate and differentiate (Sakurai et al., 2023). Due to the remarkable potential of HSCs, numerous investigations have been conducted to cure blood systemic illnesses by specifically targeting HSCs. Among these, HSCs transplantation is a treatment option for individuals with β-thal major who depend on blood transfusions (Wu et al., 2019).

It is crucial to comprehend the mechanism causing HSCs destruction because it has a bearing on the production and functionality of blood cells. According to recent research, ferroptosis has an effect in HSCs damage. Zhao discovered that when MYSM1 is faulty, HSCs exhibit elevated levels of oxidative stress and iron metabolism abnormalities by examining proteins linked to iron metabolism and measuring total ROS levels. The damage to HSCs that results in MYSM1 deficiencies is caused by ferroptosis. Ferroptosis inhibitors have also been shown to ameliorate HSC deficiencies in MYSM1 function loss (Zhao et al., 2023). There has also been revealing on a study on ferroptosis in HSCs. The researchers demonstrated that erythrocytes production is aberrant in alas2-oralad defective embryos using a zebrafish model. By upsetting iron homeostasis, heme-deficient proerythroblasts cause ferroptosis in hematopoietic stem and progenitor cells (HSPCs). Furthermore, ferroptosis inhibitor therapy can reverse the abnormalities in HSPCs(Lv and Liu, 2023). The above studies have shown that damage to HSCs is associated with ferroptosis, which involves iron metabolism and oxidative stress.

### 3.3 Ferroptosis in IE related diseases

In addition to β-thal, IE also happens to be present in various hematological disorders, including congenital dyserythropoietic anemia, hereditary sideroblastic anemia, and anemia in acquired conditions such myelodysplastic syndrome (MDS) (Cazzola, 2022). Although there are no studies showing that ferroptosis is involved in the, IE of β-thal, researches have shown that ferroptosis is participating in other hematological diseases present with, IE.

X-linked sideroblastic anemia is a disease in which mutations in the erythroid-specific 5-aminolevulinate synthase (ALAS2) gene cause excess iron accumulation and, IE. The researchers constructed a model that introduced the ALAS2 missense mutation erythroblasts derived from human cord blood, in which enhanced BACH1 expression was emerged, leading to increased susceptibility to ferroptosis (Ono et al., 2022). Liu established a mouse model of aplastic anemia with iron overload and discovered that via stimulating the Nrf2/HO-1 and PI3K/AKT/mTOR pathways, panaxadiol saponin suppressed ferroptosis in these mice (Liu W. et al., 2023). Furthermore, the IE-related sickle cell anemia pathogenesis involves a point mutation in the β-globin gene, like the pathophysiology of β-thal (El Hoss et al., 2021). By controlling the levels of l-2-hydroxyglutarate (L2HG), Nrf2 causes ferroptosis in sickle cell anemia (Xi et al., 2023). Therefore, ferroptosis is present in these blood system diseases associated with, IE.

## 4 Ferroptosis and IE in β-thal

### 4.1 Hypothesis of ferroptosis in β-thal

We hold the view that ferroptosis interacts with, IE in β-thal. As previously mentioned, ferroptosis performs a role in several hematological disorders where, IE is present, and, IE is a pathogenesis associated with β-thal. Iron overload is a major complication of β-thal and a critical link in ferroptosis. Furthermore, IE and ferroptosis both exhibit an imbalance in ROS in β-thal. Consequently, there is cause for concern that ferroptosis and β-thal are associated through, IE, with iron metabolism and oxidative stress serving as the specific mechanisms.

### 4.2 Potential mechanism

On the one hand, ROS imbalance may be an intermediate link in the interaction between ferroptosis and, IE in β-thal. The specific mechanism by which ROS imbalance leads to ferroptosis has been described in section 3.1.2. Vitamin E, an antioxidant, has been shown to inhibit cells from undergoing ferroptosis (Su et al., 2020; Wu et al., 2021). The influence of ROS imbalance in, IE of β-thal is also described in section 2.2 above. As well as when patients with β-thal were exogenously supplemented with vitamin E, researchers found that antioxidants could mitigate oxidative stress in erythrocytes through multiple targets, thereby impacting iron overload (De Franceschi et al., 2013). Consequently, both ferroptosis and, IE are closely related to oxidative damage in the body.

On the other hand, iron overload may also be an intermediate link in the interaction between ferroptosis and, IE in β-thal. Wang’s study showed that ferroptosis was observed in murine models of hemochromatosis, which is a disease related to iron overload (Wang et al., 2017). Iron overload is an important complication in individuals with β-thal, so the observation of ferroptosis in iron overload diseases provides indirect support for the hypothesis that ferroptosis is involved in β-thal. The specific mechanism of ferroptosis caused by iron overload is described in section 3.1.1 and iron overload is widespread in patients with β-thal. Based on the need for transfusion therapy, β-thal can be classified as transfusion-dependent β-thal (TDT) and non-transfusion-dependent β-thal (NTDT) (Crisponi et al., 2019). There are three categories of β-thal based on how severe the disorder is the β-thal major, β-thal intermedia, and the β-thal carrier condition (Origa, 2017). Several factors have been identified as the pathogenesis of iron overload in β-thal. Firstly, excessive iron intake is caused by multiple blood transfusions in TDT patients, which require blood transfusion therapy. Secondly, excessive iron is absorbed into the circulation through the intestine in β-thal intermedia (Musallam et al., 2021). Thirdly, iron overload is caused by, IE that leads to hemolysis in patients with β-thal. Hemolysis of erythrocytes contributes to the iron release from hemoglobin, which causes iron overload. Porter demonstrated that patients with β-thal treated with Luspatercept, a drug used for managing, IE, can ameliorate iron overload (Porter et al., 2019). This finding further substantiates the role of, IE in causing excessive accumulation of iron. Fourthly, Hepcidin starvation explains phenomenon that iron overload in patients with β-thal who have not been treated with blood transfusion. Hepcidin is an essential peptide hormone comprising 25 amino acids that is synthesized and secreted by hepatocytes. Hepcidin prevents iron overload by reducing serum iron concentration through binding to the Ferroportin (FPN) on the basolateral aspect of the intestinal epithelium and the plasma membrane of macrophages (Camaschella et al., 2020). Several studies have substantiated that enhancement of Hepcidin activity in a murine model of β-thal can effectively mitigate, IE (Cazzola, 2022). It has been suggested that Bone Morphogenetic Proteins 6,2(BMP6,2) and IL-6 can upregulate Hepcidin, while FKBP12 and transmembrane serine protease matriptase 2, encoded by TMPRSS6 can inhibit Hepcidin (Camaschella et al., 2020). They are regulated by anemia, hypoxia, and inflammation (Nicolas et al., 2002). IL-6 upregulates Hepcidin via the IL-6R-JAK2-STAT3 signaling pathway. In patients with β-thal, Hepcidin is mainly regulated by GDF15, erythroferrone, and TfR1. In β-thal, elevated EPO stimulates erythroblasts to secrete erythroferrone, which hinders Hepcidin through the BMP-SMAD pathway, leading to iron overload. Hepatocyte TfR1 interacts with HFE to inhibit the secretion of Hepcidin (Xiao et al., 2023). GDF15 is thought to contribute to the inhibition of Hepcidin secretion (Tanno et al., 2010). However, the mechanism of GDF15 in regulating Hepcidin needs to be further investigated (Srole and Ganz, 2021).

In conclusion, iron metabolism and oxidative damage-mediated ferroptosis are not independent processes but are interrelated processes. Iron as a redox-active metal has the ability to produce ROS, not only via the Fenton reaction but also through REDOX reactions occurring during the conversion between Fe^3+^ and Fe^2+^(Thévenod, 2018). The mechanism by which iron exerts its influence on the body is inextricably linked with the antioxidant system. Regarding how iron overload and imbalance in ROS homeostasis affect, IE in patients with β-thal, the current research suggests that iron overload leads to an imbalance in ROS homeostasis, which in turn causes oxidative damage to erythroid cells. Furthermore, the dysregulation of Hepcidin and Ferritin expression in ferroptosis has implications for, IE. In a mouse model of β-thal, reducing, IE can be achieved by enhancing Hepcidin activity or inhibiting Ferritin function to restrict iron production (Jiao et al., 2022). In addition, decreased expression of GPX4 in ferroptosis can affect, IE through modulation of oxidative and antioxidant systems. Vuren revealed that the relative deficiency of GPX4 disrupts mitophagy, which subsequently leads to the failure of reticulocyte maturation and affects, IE (van Vuren et al., 2020).

## 5 Conclusion and future perspectives

In brief, β-thal is a prevalent monogenic genetic disorder with a global impact. Despite extensive research on the pathogenesis and treatment of β-thal over several decades, the underlying mechanism of, IE in this condition remains elusive. IE can trigger iron overload, and ferroptosis, a regulated cell death process triggered by iron overload, has the ability to potentially modulate, IE. Consequently, a close interplay exists between, IE, iron overload, and ferroptosis in the circumstances of β-thal. This review summarizes certain interactions between, IE and ferroptosis in β-thal and proposes an innovative path for research that could enhance the anemic symptoms of β-thal.

Nowadays, the two primary techniques for identifying ferroptosis are direct examination of mitochondrial morphology using transmission electron microscopy, and indirect detection of lipid peroxidation-related markers such as Fe^2+^, ROS, GSH, malondialdehyde (MDA), LPO, and GPX4. The lack of specificity of indicators hampers the determination of ferroptosis in iron overload induced, IE. Therefore, it is imperative to identify specific biomarkers associated with ferroptosis. Although it is clear that iron metabolism and ROS metabolism participate in the interaction between, IE and ferroptosis, the specific molecular mechanisms of iron or ROS metabolism are still unclear, and the common molecular mechanisms of iron and ROS are also not clarified. This suggests that the mechanism of the interactions between, IE and ferroptosis in β-thal requires further investigation. In conclusion, current treatments for patients with β-thal do not fully relieve their symptoms, and there is great potential that the study of the interactions between, IE and ferroptosis in the treatment of β-thal.
